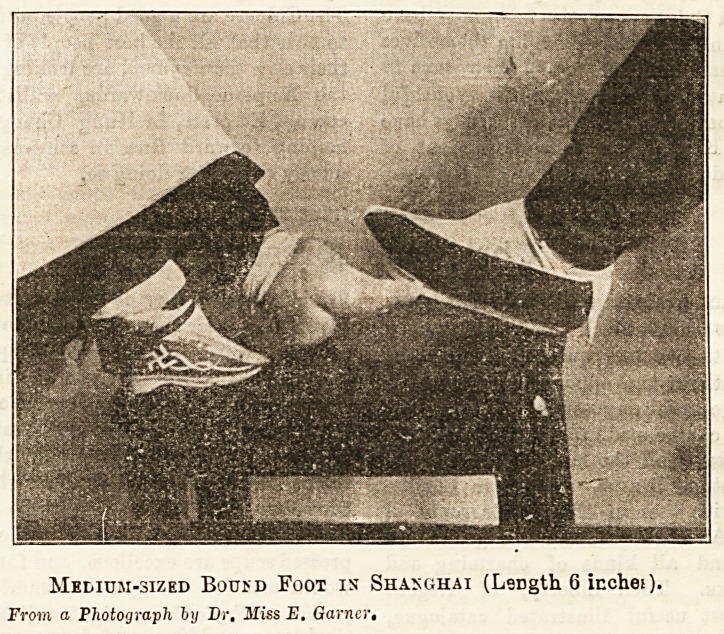# "The Hospital" Nursing Mirror

**Published:** 1898-12-17

**Authors:** 


"The Hospital" Nursing Mirror.

Being the Nursing Section of "The Hospital."

[Contributions for this Section of "The Hospital" should be addressed to the Editor, The Hospital, 28 & 29, Southampton Street, Strand, London, W.C., and should have the word "Nursing" plainly written in left-hand top corner of the envelope.]

### News from the Nursing World.

### THE QUEEN AND THE SOUDAN NURSES.

The Queen was fortunately able to bestow the decoration of the Royal Red Cross upon two of the three nurses recently awarded this distinction for service in the Soudan. Miss Sarah Emily Webb, superintendent of the Army Nursing Service, and Miss Elizabeth Low Geddes, of the National Aid Society, were presented to Her Majesty at Windsor on the 8th inst. for this purpose by the Dowager Lady Southampton (the Lady-in-Waiting in attendance). The Princess Henry of Battenberg was present on the occasion.

### OUR CLOTHING DISTRIBUTION.

Those of our readers who have not already done so will greatly help us if they will send in their contribution to our Clothing Distribution at once. We are within hail of Christmas Day, and the parcels when received have to be sorted, repacked, and sent out. The fund at the disposal of the editors has been expended to great advantage at Messrs. Garrould, Edgware Road, a firm which has kindly added a liberal discount to our bounty. A nice package from Nurse Wilson has also arrived, and next week we hope to announce the destination to which these contributions have been sent.

### TRAINING NURSES AT LEWISHAM INFIRMARY.

The whole system of training nurses has been carefully studied at Lewisham Infirmary. Definite courses of instruction are now given by the matron, medical superintendent, and assistant medical officer, interspersed with frequent examinations, culminating in a final one at the end of the three years, conducted by Dr. Arkle, of Charing Cross Hospital, as outside examiner. Special arrangements are then made by which nurses who desire it may take up a course of maternity nursing, and prepare for the L.O.S. certificate, the small lying-in department of the infirmary giving them special facilities in this respect. Every three months the sisters of the wards send in a record of marks for the conduct, temper, cleanliness, accuracy, and efficiency of each probationer under her instruction, which reports are considered in the examination. The majority of the sisters have been trained in London hospitals, and the matron herself was trained at St. Bartholomew's and, in addition, had a couple of years' experience of poor-law work at Chelsea Infirmary. In consequence of these satisfactory arrangements there is no lack of candidates seeking training.

### TO PENSION FUND NURSES.

Last week we acceded to a request of several members of the Royal National Pension Fund for Nurses that we should help in arranging a wedding gift to Miss Burns, daughter of Mr. Walter Burns, late chairman of the Pension Fund. In the interests of nurses we stipulated that the contributions should be limited to sixpence, and that the gift should take the form of a congratulatory address. The list will be closed on the 24th inst.

### NEW NURSES' HOME AT BIRMINGHAM.

In opening the new nurses' home in Moseley Road, Birmingham, the Lord Mayor put his finger on the weak spot of most medical charities, namely, the small number of subscribers in comparison with the population. That the list in a large and wealthy city as Birmingham should only reach 372 is humiliating, and that Birmingham should only be one place among so many is distressing. The new home, the opening of which on the 9th inst. brought many of the more influential residents together, is the second house acquired by the District Nursing Association, and is part of a wider scheme which embraces the systematic nursing of the whole city. Money was provided by a grant from the Diamond Jubilee Fund and by private subscriptions. Miss Waller is the lady superintendent, and a staff of eight nurses will work under her.

### BELFAST NURSES' HOME.

There have been numerous changes in the staff of the Belfast Nurses' Home during the last year, but that is only what may be expected out of 92 nurses. The Home Sister, Miss Clutterbuck, left for Bolton, and the night superintendent, Miss Green, has taken her place; whilst Miss Warr, of King's College Hospital, has stepped into Miss Green's shoes. The private staff has been removed to 17, College Square East, on account of increasing pressure on accommodation, and this new extension is under Miss Newman's superintendence, with Miss Rowan as housekeeper and matron The latter has had nine years' experience in this service of the association, and is much liked. According to an arrangement made last December all the certificated nurses received a 10 per cent. bonus on their fees, and one nurse has been superannuated. The whole question of age-provision for nurses has been brought up by this nurse's resignation, and will be met either by an arrangement with the Royal National Pension Fund or privately, as may appear most advantageous.

### LADY FORSTER AND WALSALL NURSES.

On the 2nd inst. a large and influential company assembled in the Lecture Hall of the Science and Art Institute at Walsall, on the occasion of the opening by Lady Forster of the new home for the Walsall Nursing Institute. The Mayor took the chair. The report of the Lady Superintendent was read, Captain Cozens explained the financial position of the institute, Mr. J. R. Cooper gave a history of the inception and working out of the scheme, and Lady Forster expressed her pleasure at being amongst her friends again, and said that she hoped the institute would be as successful as it ought to be. Our readers already know that this institution was founded by aid of the balance of £2,500 which remained out of the fund collected for the Jubilee celebration. Miss Eyre is the lady superintendent, and she and three nurses have already taken up their posts at the new home at 40, Bradford Street.

### OLD AND NEW MATRONS AT HEREFORD.

We chronicle this week the appointment of Miss Elphick as matron of Hereford General Infirmary, and it is also pleasant to note the honours accorded to Miss Sharp upon her retirement from this office. On the 6th inst. the nursing staff arranged a little festival in their sitting-room, at which Miss Russell, Miss Fish, and Mrs. Attwood-Mathews were present, and gave to their outgoing matron a silver tea service. Mrs. Attwood-Mathews, in making the presentation, expressed the appreciation which Miss Sharp has won during a period of twelve years spent in the careful performance of her arduous duties.

### THE "DAVID LEWIS NURSES' HOME."

The large nurses' home which is the first part of the David Lewis Northern Hospital, Liverpool, to be finished was informally opened recently. It stands at the corner of Great Howard and Leeds Streets, and is a commodious building of red brick. It contains 63 bed-rooms, sitting-rooms for the lady superintendent, sisters, and nurses, a class-room for instruction in cookery, with a full allowance of bath-rooms, &c.

### THE EARNINGS OF THE TRAINED NURSE.

The report of the Glasgow and West of Scotland Co-operation for Trained Nurses read at the annual meeting recently held at 18, Sardinia Terrace, Glasgow, possesses an interest apart from the satisfactory record of growth and prosperity of the society, inasmuch as it gives the earnings of the individual nurse in that district. They have been in full work during the year, and the highest sum earned by one nurse was £85. Those who permitted themselves a month's holiday received from £60 to £80. This is not a fortune, neither is it a pittance; still it is enough to provide a comfortable maintenance in the present and, with reasonable forethought, a competence in age—blessings not to be despised in any age of the world's history.

### THE NURSES' NEEDLEWORK GUILD.

Nurse Theobald, 4, Bolsover Street. W., has just despatched her second year's distribution of clothing fashioned by the members of the Nurses' Needlework Guild. Four hundred and seventy garments of all kinds have been sent in parcels of 50 to needy institutions.

### NURSING AT MANCHESTER AND SALFORD.

The principal feature of the annual report of the Manchester and Salford District Nursing Association, which was read at the Manchester Town Hall on the 7th inst., is the critical financial condition of the society; a state that threatens to cripple the district work of the institution by making it imperative to discontinue the services of one nurse. In a year's expenditure of £6,251 there is a deficit of over £560. The more serious aspect of the matter is that this is in face of a demand for more workers. We note an improvement in the internal working of the association, and an acquisition; the latter is a new home for the nurse at Salford, which will be ready for occupation in the spring; and the former is the opening of a branch of the private staff conducted on co-operative principles. Sir F. F. Adam has consented to become president of the association in place of the late Archdeacon Anson. In seconding the resolution moving the acceptance of the report, Mr. Rathbone mentioned that in Liverpool visiting nurses for paying cases had been added to the district staff, and arrangements made for some of their nurses to visit regularly the Board schools.

### SHORT ITEMS.

The new matron of the General Civil Hospital, Hong Kong, has chosen an assistant, by desire of the authorities, to help to found a training school for nurses, where Eurasian and Chinese women will be received as probationers. The heads of the department will probably be English-trained sisters. A portion of the Jubilee funds has, it appears, been voted to the erection of adjuncts to this hospital, amongst which is a nurses' home.—Messrs. Fred. C. Everill and John L. Wagstaff gave an evening entertainment on the 5th inst. for the patients of the Cancer Hospital (Free), Brompton, London, S.W., which was very successful. Mr. Dan Leno sent a cheque for £5 5s. The programme consisted of dramatic and musical sketches, songs, &c.—The Christian Commonwealth Home Sunbeam League gave its fifth annual sale of work and entertainment in aid of funds to maintain a cot at the East London Hospital for Children, at the West London Tabernacle Hall, Notting Hill, W., on the 13th inst.—There is to be a parish nurse for Mexborough, who is to work on strictly "self-help" lines. The following regulations give scope for much discussion, present and future: Patients must be recommended by a subscriber, and the recommendation signed by a medical man. Working folk may contribute one penny a week, and be nursed free upon a recommendation signed by the doctor.—The Stockport Sick Poor Nursing Association has been established seven years, and now the people for whom it was founded eagerly avail themselves of the advantages it offers, although it took some little time at first to win their confidence. Nurses Garner and Tofts are much liked.—The nursing arrangements at the Albany General Hospital, Grahamstown, are reported to be very good, though the pressure of work renders it desirable that another nurse should be added to the staff. The probationers are well spoken of as conscientious and capable workers.—Invalids visiting Florence may be glad to know of an establishment whence they may obtain trained English nurses and accommodation. Miss Violetta Fasulo's private nursing home, 7, Via Rondinelli, Florence, has been recommended to us. English nurses can also obtain board and residence at this home.—The present matron of the Peterborough Infirmary and Dispensary (Miss Pedder) has, with the sanction of the committee, withdrawn her resignation.—On January 20th, 1899, the new nurses' home in connection with the Chelsea Hospital for Women, Fulham Road, S.W., will be opened by the Duchess of Albany.—The twenty-ninth annual course of entertainment at the National Hospital for the Paralysed and Epileptic for the in-patients began on the 1st inst. They take place fortnightly, omitting one during Christmas-week. Different ladies and gentlemen are responsible for the programmes, which occupy from seven to nine p.m. on their respective evenings.

### Chinese Foot-Binding.

By Mrs. Archibald Little.

Foot-binding is not a mark of rank in China. In the districts where it prevails all women are bound, even to the beggars by the roadside, with the exception of the slaves, and in the East of China the boat-girls. In Manchuria, where the women work in the fields, they do so kneeling on their knees in the heavy clay soil, being incapable of standing. And few pictures can be more pathetic (or less beautiful) than that of these poor women doing rough field labour for the barest pittance, patiently kneeling in order to get husbands. No Chinaman will ever say that it is in order to keep the women from being gadabouts that their feet are thus mutilated. Chinese confine themselves to saying "It is *hao kan*," or "good to see," and till of late no girl with natural feet had a chance of getting a husband, the first question of a young man with regard to his proposed bride being, "What is the size of her foot?" Then the middle-woman produces a fairy shoe, and the young man carries it about in the folds of his big sleeve—the Chinaman's pocket—and shows it to his young men friends with exultation. Very likely it is not the shoe of his promised bride at all, and only last year I heard of a bride being returned to her family for another year's bindng, her foot not being found small enough.

The age for beginning binding varies in different parts of China from five to eight. It takes about three years. The length of the bandage, I believe, also varies from two to five yards. It is about three inches wide. The mother, leaving the great toe free, binds the child's other toes as tightly as possible underneath the sole of the foot, so as to bring them out at the other side, where they eventually appear looking like the white worms found in the garden after rain, if they do not mortify and break off in the process. The first year is devoted to thus narrowing the foot, the next two to shortening it, which is much more painful. The bandages are so placed as gradually to draw the fleshy part of the sole of the foot and the heel so closely together that a half-crown piece could with ease be hidden in the cleft thus produced between the two. Until this can be done no foot is considered bound at all. It is commonly reduced to a length of 5½ inches from the tip of the great toe to the end of the heel, but a lady once called upon me whose foot actually did not quite measure 2½ inches. I wanted to take off her stocking to see what the little deformed lump really looked like, but the discharge that I could not but see soiling her stocking, when she took off her shoe, sufficiently accounted for her unwillingness to oblige me.

In all Chinese pictures of foot-binding the mother is depicted with a big stick, dragging along a screeching, refractory little girl. And it is said mothers habitually sleep with big sticks by their side with which to beat the little girls should they disturb the household by their wails at night. But in many cases the poor little girls are put to sleep by themselves in outhouses. When the bones of the foot are too elastic, resuming their natural position each time the bandages are withdrawn, a big wooden mallet is used to smash the small bones, and thus hasten the process. A lady, head of a school, herself told me that on one occasion when all the mothers were bringing their little daughters new shoes, she herself saw one mother, finding the shoes she had brought did not fit, take up a large pair of scissors from the table and there and then cut off a bit from the child's foot. Of course, that woman was forbidden ever to enter the school again. But the hardening effect on the hearts of the people of China from inflicting and witnessing the sufferings of their little girl children are incalculable. Most of us look back to a mother who was always so kind and tender; the Chinese child looks back upon a mother who inflicted years of torture and beat her little girl when she cried.

Medium-sized Bound Foot in Shanghai (Length 6 inches).

*From a Photograph by Dr. Miss E. Garner.*

After binding the child is forced to walk about to prevent mortification at once setting in, and then the process is repeated morning and night, each time drawing the bandages tighter, during all those dreadful three years. The child's only relief is by hanging her feet over the edge of her wooden bedstead so as to stop the circulation, and sometimes she is given opium. By day she hobbles about leaning on a stick taller than herself, with great black lines under her eyes, and a curious pallor on her cheeks that I have never seen except from foot-binding. Sometimes she is carried about on her father's back. Not uncommonly she sits and cries.

The Chinese saying is that each pair of bound feet has cost a *kang* or big bath full of tears, and that one in ten dies of foot-binding or its after effect. When I repeated this to a saintly Italian mother superior at Hankow at the head of a very large school and foundling asylum, she said with tears in her eyes: "Oh, no, no! that may be true of the coast towns, but not here. Here it is more than that." I have never spoken with any doctor in practice amongst Chinese who has not on undoing the bandages seen the feet either drop off or only fastened by a filament, so that they had at once to be cut off. A doctor at Chungking told me he had seen three such cases, and besides those had two women brought to him paralysed, whom he had cured simply by massage and undoing their bandages. A doctor at Nankin told me of a child whose grandfather was an official, her father a teacher. She was about seven years when her feet were two black masses of corruption. Her relatives would not allow the feet to be amputated, so in a few months they dropped off. They were a long time healing, as the skin was drawn back from the bone; they soon became sore again on her going home, she became weaker and weaker, and after a year and a half of suffering died. If every foreign doctor can relate cases like this, how many must there not be away from foreign aid? Lady doctors tell us it is rare to see a woman patient with all her toes in Shanghai, and several of them are of opinion that stumping about on the heel, necessitated by the mutilation of the feet, so jars the woman as to cause womb complaint of some kind, as they notice that in the districts where binding is universal all women patients suffer from something of the kind, whereas where binding is rare womb complaints are also rare. Sufficient data have not been collected yet to establish this. It is, however, clear to a most casual observer that the ladies of China have wretched health, their legs are reduced to the size of the leg-bones, they have no hips, and consequently, to keep their skirts and trousers from dropping off, tie strings round their waists so tightly as often quite to cut into the flesh. Beyond suckling children and embroidering shoes they have no occupations. They smoke, in the west opium as well as tobacco; they have their hair dressed, they talk, and often go to and give ladies' dinner parties. They seem exceedingly grateful for the efforts foreign ladies have been making to free them from binding, which they all say "is of no use at all, and you don't know how it hurts." Influential Chinese societies have now been started throughout the country, fathers pledging themselves not to bind their daughters' feet nor marry their sons to bound-foot girls. The movement amongst the youthful literati is very strong against binding. Three Viceroys have denounced it, also Duke Kung, the lineal descendant of Confucius. Although we do not yet know how far the reactionary *coup d'état* of the usurping Empress of China may arrest the party of progress. She has cut off the heads of all the leaders that she could catch; the others are hiding for a time. Any small contributions I would thankfully receive for the Tien Tsu Hui (Natural Feet Society), which I founded about three years ago, and of which I am organising secretary. But I would recommend anyone interested in the welfare of China, and with pounds rather than shillings to spare, rather to send them to James Wenley, Esq., Bank of Scotland, Edinburgh, hon. treasurer of the Society for the Diffusion of Christian and General Knowledge in China. The Chinese are so eager for the publications of this society, that when I left China in June last they were buying some of their books at five times the published price in the remote west, and it is the spread of knowledge that is arousing the youth of China not only against the barbarous practice of mutilating their women's feet, but also against the cramping of their own minds by confining them to the learning by heart of the ancient classics of their country. It is a new China we shall soon have to face, whether we like it or not, and it depends in some measure upon all of us how soon the age of mutilation passes away, and whether the new China be materialistic, as Japan, or not only enriched by steam and rails and modern appliances, but enlightened by that higher Wisdom that alone gives peace and hope. The Chinese are a good people, and it is specially interesting to note that all the best people of China, those esteemed by their own countrymen, are looking to England for help from the Emperor downwards, whilst it is the reactionary, corrupt Empress, Li Hung Chang, and all that set that is helping forward Russian schemes, probably with a good money reason for doing so.

### Christmas Presents.

The shops are brighter and prettier than ever this year, and since no warning voice could check the flow of money towards Christmas presents, we feel quite sorry for the country cousins who are without the opportunity of making what they mean to spend go as far as possible. The smallest sum spent with discretion can be made to afford pleasure to all kinds of recipients. Nearly all the large drapery establishments have their Christmas Bazaar. Nurses in London are, no doubt, familiar with that of Messrs. Garrould of Edgware Road, already. At these bazaars the grown-ups and the children alike find all kinds of charming and useful little knick-knacks. Then Liberty, of Regent Street, has issued a most useful illustrated catalogue, which country nurses can procure and find something therein to suit all tastes. We notice especially the daintiest of needle-cases, scent sachets, work-bags and baskets, handkerchief cases, and footstools. Of course, there are endless varieties of things purely ornamental, but we most of us have too many of these dust traps already. Messrs. Houghton and Gunn's catalogue (162, New Bond Street) will surely reveal that difficult article, a present for a male friend. Their patent envelope sealer is quite a novelty, and their dog call and knife combined is most useful. The Art Embroidery showrooms (J. Harris and Son, 25, Old Bond Street) have a delightful show of Christmas gifts; and in New Bond Street also, at Messrs. Mühlen's, No. 62, is to be found the very best Eau de Cologne, the well-known 4,711. What more welcome present than this? They have other charming perfumes, especially their own, such as Rheims violets and Maréchal Niel. These perfumes suggest another popular gift, especially in the winter months. We allude to pocket handkerchiefs, which can be found no better nor cheaper anywhere than at Messrs. Robinson and Cleaver, in Regent Street. They will sell nice handkerchiefs as low in price as 2s. 6d. the dozen, or as high as to please the most lavish giver. At their shop, too, is to be found all kinds of useful articles, warm blankets, cosy quilts, and warm underclothing. For the little ones, if toys are not suitable, Cadbury's excellent chocolates are always safe and delightful. Most confectioners sell neatly arranged Christmas puddings which require heating only, and these are very acceptable to the lonely possessor of rooms who cannot join friends on Christmas Day. Nowadays there are numerous appetising forms of preparing food in portable form. Some of the compressed soups are excellent, and foremost amongst these we would mention Maggi's Consommé. A box of these and a packet of Fry's Cocoa might well be added to the gift of the Christmas pudding and forms an agreeable and far from costly present to many who have neither the means nor opportunity to secure extra comforts at Christmas time, when every Briton regards it as his right to feast and rejoice. Then we must say one word respecting Christmas cards and calendars this year. They are as numerous and varied in design as ever, and the time-honoured custom evidently is not moribund as yet. Every bookseller, stationer, and most drapers present a goodly show. We saw a very dainty little card in scarlet, white, and gold at Messrs. Speaights, the photographers, at 178, Regent Street. Indeed, turn where you will, talent and ingenuity are everywhere visible turned to the service of fulfilling the demands of Christmas givers.

### Presentations.

An interesting ceremony took place at the new nurses' home of the Union Infirmary, Keighley, on the 1st instant, when Nurse Griffiths, of Wrexham, was presented with a clock by Miss Heath, the superintendent nurse, and a silver preserve dish and spoon by the other nurses and officers. The presentation was made on Nurse Griffiths leaving the infirmary, and in testimony of the high regard of her fellow workers.

### A Christmas at Malta.

On the Christmas morning of which I write Valletta was looking quite beautiful with its bright sunshine and with its high white buildings gleaming against the azure blue of a cloudless sky. The bells from all the numerous churches had been ringing gaily from early morning, and the Maltese were crowding out of the churches as we walked along Strada Merchants to the Military Hospital.

The hospital is of great historical value, being the same that was built by the knights of St. John. At the gateway a sentry stands, and on one side is the prisoners' ward, whilst on the other is the sentries' room.

Passing into a large square paved with stone one comes upon an old well, and at the right hand side are also the sergeant-major's quarters, the dispensary, and the stores, above which is the hospital for soldiers' wives and children. At the corner are the rooms of the quarter-master of the Medical Staff Corps and the sleeping apartment of the orderly medical officer. Below the principal medical officer's offices is an archway leading to the medical officer's library, and beyond that again another archway the entrance into a long corridor, upon which open some of the sick wards. At the end of this corridor is a broad staircase leading to other wards. We will go down the long corridor until we reach the large ward, which we will enter.

This ward is the longest room in Europe without having any pillars to support it, and there are windows only on the sea side, which are about five feet from the ground with steps going up to them. The floor is of white stone, and on the morning of this Christmas Day there were no decorations on those high whitewashed walls. Only on the three six-feet barrack-room tables were some green plants in pots, and some beautiful flowers in vases which the nursing sisters had arranged. The beds on each side were all occupied by soldiers suffering from fever, this part of the ward being set apart for all the serious cases. It made little difference to these patients what day it was, but one or two of the fifty inmates were looking at Christmas cards.

The benches on each side of the tables were unoccupied, and it was the barest ward that I ever saw in my life, the very high roof of dark teak giving it a solemn appearance. But passing through a door in a barrier made by a wall seven feet high, one reached the centre division, and here there were pictures hanging on the walls, and off this part is the lovely little old chapel, which is most interesting. The tables in this division were all put together and the convalescents, dressed in blue flannel, were helping the sisters to decorate them with flowers, oranges, and fruit. The nursing sisters looked very bright with their grey dresses and white aprons and caps and scarlet capes. They were very busy carving the roast beef for the men, the dinner being at noon, and it consisted of roast beef, vegetables, and plum pudding (or duff as the soldiers call it), and beer. But tea is the meal to which all were looking forward. This took place at half-past three, and some of the wives of the officers came and helped to distribute it. There were cakes, fancy biscuits, bread, butter, and jam. Fruit and crackers were also on the tables, which looked very pretty and were well covered with good things. All the convalescents seemed very happy, but they were kept quiet on account of the fever patients on the other side of the wall, who, poor things, had to be fed on milk only. One lady had kindly brought a little bunch of flowers for each of them, which they enjoyed, violets being the favourites.

After tea was over everyone went upstairs to one of the convalescent wards, where there were bran pies containing a present for each man. Each patient received also a pipe and an allowance of the weed they all love so much—tobacco. Then they were allowed to smoke until nine o'clock and sing. The medical staff corps orderlies had a festival dinner in their barrack-room, which was decorated with coloured papers and different mottoes. They had a grand dinner of roast turkey and roast beef, plum pudding and all sorts of good things, with as much beer as they liked from the canteen.

The nursing sisters, of course, were very busy, as all the sick had to be nursed, and one of the sisters never left them; nevertheless, all the ladies went into their sitting-room at the sisters' quarters—which are at the left hand of the square. It was the prettiest room in Malta, so tastefully arranged and of such a quaint shape.

### Our American Letter.

The Waltham Training School for Nurses has seen several changes during the last twelve months. Miss Harriet Rainsford has been appointed Lady Superintendent; a batch of its graduate nurses have dared the wilds of Klondyke, and others have enlisted under the Victorian Order of Nurses, Canada, founded by Lady Aberdeen. A pretty nursing home of three stories, containing accommodation for 18 nurses, is in process of erection in the grounds of the Chester City Hospital, Westchester, Pa. The big kitchen will be also utilised as a cooking school. Typhoid fever has attacked several of the nurses employed in the army during the late campaign; nevertheless, this does not deter other nurses embarking on the same service, and some from the Clarinda Hospital, Iowa, have gone to Fort McPherson and Chickamauga, some from other schools to Jacksonville, Fla., and Fort Monroe. Dr. Anita McGee has appointed Miss Rose Kane, a graduate of the Homœopathic Training School for Nurses, Reading, Pa., an army nurse. She will be put in charge of one of the Government hospitals.

### Where to Go.

Art Embroidery Showrooms.—Our London readers must not forget to visit J. Harris and Sons' Christmas sale and exhibition of art needlework at 25, Old Bond Street, W. The materials are beautiful and attractive, besides having the merit of being durable. The designs are also novel and pleasing, and the setting of the work, lace, trimmings, &c., is harmonious and artistic. Country cousins may have parcels sent to them for selection upon receipt by the manageress of either deposit or reference.

The London Hospital.—The Christmas entertainment at this hospital will take place on the 24th inst, and will probably be as popular as those in previous years. On Christmas Day itself visitors will be admitted to see the patients from three to five o'clock.

### Death in Our Ranks.

Quite a gloom has been cast over the City Hospital, Edinburgh, by the death of Miss Janet Thomson—a valued and beloved nurse—from typhus fever, contracted in the discharge of her duties. A sharp outbreak of this disease occurred in the city during the latter part of November, and although successfully grappled with by the authorities, resulted in the loss of a nurse who has been attached to the hospital for the last twenty years, and who has been at work in the city for thirteen years. Nurse Thomson was buried on November 30th at the Calton New Burying Ground, several influential public men and as many of her fellow-nurses as could be spared attending the funeral.

The lady superintendent and staff at the Fir Vale Infirmary, Sheffield, deeply regret the death of C. S. Fitton, a probationer of ten months' training, from typhoid fever, on November 21st.

### Travel Notes.

By Our Travelling Correspondent.

Rules in regard to Correspondence for this Section.—All questioners must use a pseudonym for publication, but the communication must also bear the writer's own name and address as well, which will be regarded as confidential. All such communications to be addressed "Travel Editor, 'Nursing Mirror,' 28, Southampton Street, Strand." No charge will be made for inserting and answering questions in the inquiry column, and all will be answered in rotation as space permits. If an answer by letter is required, a stamped and addressed envelope must be enclosed, together with 2s. 9d., which fee will be devoted to the objects of the "Hospital Convalescent Fund." Any inquiries reaching the office after Monday cannot be answered in "The Mirror" of the current week.

### II.—HINTS TO TRAVELLERS.

I shall occupy our space for the next two weeks with prosaic details as to the cost of the journey, outfit, luggage, exchange of money, circular notes, &c. It will be neither interesting nor exciting reading, but such matters are all important; and if unaccustomed travellers would make themselves thoroughly conversant with them foreign travel would be robbed of the nightmares that haunt the pillows of the inexperienced.

### Quickest Route to the South.

First, we will take the journey to the South of France, where delicate people, or those disliking the rigours of an English winter, betake themselves like the swallows at the first sign of cold weather. The quickest route is by Dover, Calais, Lyons, and Marseilles, and costs, first class, £7 14s. 9d.; to Nice, and second class, £5 5s. 8d. There is a cheaper route *viâ* Newhaven and Dieppe, and thence by Paris, Lyons, and Marseilles, first class, £6 12s. 4d.; second, £4 11s. 8d.; making a difference, roughly speaking, of £1. This gain, however, is only in appearance, for a delicate person would be compelled to sleep in Paris, having already had so fatiguing a journey, and being unable to leave Paris until the 8.45 train, whereas the night in Paris may be avoided in cases where the traveller is not a serious invalid, or if it is intended to take a sleeping car through.

If money is no object, and the traveller equal to it, I should advise the Dover and Calais route, taking your tickets of Dr. Lunn, Gaze, or Cook (which obviates many little inconveniences), and going through Paris without stopping. By taking Ceinture railway tickets your luggage is passed through to Nice, where it is examined. This I will explain later on.

Arrived in Paris you have time for a wash and rest, going on from the same station (the Nord) at 7.44. If it is advisable to sleep the night, do so at the Station Hotel, which is very comfortable. Going on by the 7.44 you dine on the train as it makes the circuit of Paris; the dinner is very good, price 5fr. 50c. The sleeping cars are commodious, and a great boon to those not robust. The extra expense approximates to 28fr. for each person for every 300 kilometres; this adds considerably to the expense, but the gain is so great in rest that it is often possible to go through to Nice without stopping, thereby saving the cost of hotels, &c., *en route*, so that for an invalid the two modes come to about the same cost.

### Where to Break the Journey.

It is a question to be decided individually by each person in charge of invalids how much stopping on the road is advisable. It appears to me common sense to make the following rule apply, with modifications. If the traveller is bodily weak and frail, he should stop often on the road, even more than one night, so that the harassed feeling engendered by knowing that he is again on the wing very shortly may not take hold of him. Never travel more than ten hours without a break and rest—long enough to really make up for the previous wear and tear caused by actual fatigue, and also by the unwonted excitement of the complete change of life and anticipations of the future. On the other hand, if there is not much real weakness or disease present, but rather a nervous condition, then I think it is better to go straight through or with only a night in Paris, because the excitement of moving in and out of trains, arriving and departing from hotels, and sleeping in new beds keeps the brain continually exercised and the nerves in a state of tension, and does away with the advantage of a stationary night. If several breaks are necessary let them be at Lyons, which you reach at four in the morning, and at Marseilles. At Lyons stay at the Grand Hotel Collet and Continental; at Marseilles, at the Terminus, which is attached to the station. It is expensive but convenient, and you are spared the expense of cabs.

### Avignon a Good Stopping Place.

If one break on the road is sufficient besides Paris, Avignon might be chosen instead of Lyons or Marseilles. The city teems with interest, and is well worth a stay of two or three days if circumstances permit, whereas Lyons is decidedly uninteresting—a modern commercial town, in fact. Marseilles is a fine city, and, in its cosmopolitan way, unique; but it is a fatiguing place, and too bustling and noisy to be congenial to invalids. If you stay there twenty-four hours be sure to go to the harbour, where Jews, Turks, infidels, and heretics do congregate. It is indeed a strange sight—every nationality and almost every costume is to be found there. If you elect to stay at Avignon rather than at this busy port go to the Hotel de Luxembourg.

### The Coupon System.

By using the travelling tickets of either of these firms you have a certain advantage, especially in the matter of luggage. It is a shock to the British mind, accustomed to travel with basket trunks and boxes resembling Noah's Ark in dimensions, to make the unpleasant discovery that one must pay for one's luggage on the Continent. In England, however outrageous in size and number one's impedimenta, it is very rarely that one has to pay overweight, and if so, we call heaven and earth to witness the injustice, and talk of writing to the *Times*; thus we are in the habit of launching out in the matter of luggage somewhat luxuriously, but unless you want to pay several pounds extra you must limit your requirements considerably. By using the tickets issued by Dr. Lunn, or Messrs. Gaze or Cook you can carry free, in France, 56 and 66 lbs. of luggage, according to the lines used; in Germany, 56 lbs.; in Holland, 56 lbs.; but in Belgium, Austria, Italy, and Switzerland no allowance is made, and they inexorably demand their full pound of flesh. In Italy you may take almost anything short of a boat or an elephant in the carriage with you, though the stated permissible dimensions are 19 by 10 by 12 inches. The proviso is added that all baggage must be so placed as not to inconvenience your fellow-passengers. This last clause is by no means strictly obeyed; the thrifty Italian manages to convey himself, his family, and entire stock of luggage with him, and not pay an extra centime. The results may be imagined. I well remember a journey from Venice to Florence, where every inch of the gangway and the racks was occupied. My knees nearly knocked my nose in consequence of my feet being insecurely planted on an enormous parcel of slippery exterior and angular development. The proprietor made a merit of its presence as being a great advantage to me, and addition to my comfort. When anyone wished to move, which was constantly, for Italians are the most restless of mortals, we all clutched our bags and parcels feverishly to our bosoms, and sustained many shrewd pokes and thumps from our neighbours. In spite of these drawbacks, however, Italy is a pleasant country to travel in, the people are so genial, so polite, and so gay.

REGISTRATION OF LUGGAGE.

Luggage is registered at Charing Cross straight through to Nice and other points on the Riviera if the traveller takes tickets by the Ceinture Railway; this is an immense improvement upon the old plan, when the Customs examination took place in Paris. Even if you sleep on the road your luggage will go on safely, and you will find it at your journey's end. Whenever you encounter the Customs examination abroad, remember to afford all assistance possible to the officers. It is insular, and ill-bred too, to resent anything that is the national habit of the country through which you are travelling, and if you on your part do your best to aid the harassed men who have to carry through so wearisome an affair, I think you will find that you meet with politeness and consideration.

HAND LUGGAGE.

As you will be parted from your heavy baggage be sure to arrange that you have with you all that is necessary for the night, or for several nights, if your journey is taken slowly. Besides toilet arrangements, take a warm dressing-gown if in a sleeping carriage, so as to remove dress and corsets, and thus rest really properly. It is an immense comfort to have a small air pillow to fill up hollows. Have good warm rugs, also thoroughly easy slippers. Take all your apparatus for washing and hair-dressing in a flat receptacle with compartments, that rolls up like a gentleman's shaving-case, so that you may not be hunting wildly for stray curling pins and hair strings in the corners of your dressing bag.

AVOID SUMPTUOUS DRESSING BAGS.

One word as to these elegant accompaniments. They are most inconvenient for Continental travel on account of their weight. You must always remember that there are comparatively few porters abroad, and there are wearisome restrictions that the outside porters shall not enter the station, and the inside ones shall not set foot outside; furthermore, in many places they are not permitted to enter the railway carriage at all, and, heavy or light, you must hand your bags through the window to him. All these trials warn the experienced traveller to have nothing for show, but all for convenience. I am myself the proud possessor of a gorgeous bag, which I always feel adds great distinction to its proprietor; but after travelling through Italy with it, I began to loathe its presence, and have since supplied its place with a 4s. 6d. bag, made of India matting of some sort, that locks, and weighs when empty only 6 oz.

A TEA BASKET IS ESSENTIAL.

Without a tea basket you are a lost creature on the Continent; mine has been all over the world with me, it comes in conveniently for so many occasions and for so many purposes, and on the journey, especially, you will prize it. Station tea is always a liquid horror, and the loathsomeness of it is enhanced on the Continent by the fact that they keep their tea in cardboard boxes exposed to the air, and put only an infinitesimal pinch into a vast and chilly china teapot, filling it up to the brim with water that does not boil, in which the unsoftened leaves float disconsolately. Such is Continental tea, which they regard as a medicine and confidently pronounce good for the nerves! It is often preferable to take food with you in the carriage as far as possible, for it is a scramble at the buffets, and the invalid is frequently not inclined for a meal at the only times when they are procurable. Pressed home-made meat moulds are excellent for the purpose, and small rolls which you can open and butter freshly each time are convenient, whilst strong meat extracts can be used with the help of your kettle. It is well to take a tin of condensed unsweetened milk with you, it keeps perfectly sweet for a week, and fresh milk will turn sour in a journey from the shaking and lack of air. When the water in your kettle is exhausted it is always easily renewed at the stations, the guard will always do it for you "for a consideration."

FORWARDING EXCESS LUGGAGE.

If your luggage is very voluminous, which is unavoidable if you are going to make a sojourn abroad for six months, and especially if you are to occupy apartments, it will be rather cheaper to forward the heavier boxes by the International Baggage service or Continental Parcels Express. Messrs. Gaze or Cook will arrange it for you. It will be from three to ten days on the road.

FRENCH BUT LITTLE NEEDED ON THE JOURNEY.

It is very pleasant and convenient to be able to speak French fluently, but it is not essential on the southern route to all the big places, such as Paris, Lyons, Marseilles, &c. There are interpreters, and Messrs. Gaze and Cook have agents at the stations, whom you will easily recognise by the motto on their caps; they are always most obliging and helpful, and if you find your French shaky it is much better to employ them. Why struggle for onesself when one can have someone else to struggle for one?

CARRY YOUR TICKETS IN AN ACCESSIBLE PLACE.

In Continental travelling one is continually asked for one's tickets. Put them in a small bag hung round your waist on a strap, in which should be also your money for the journey, English and French in separate pockets therein. Some rash folk are addicted to secreting tickets in a fresh place after every examination, which results in great disaster, calculated to try even the patience of a French guard accustomed to the strange vagaries of the English. I recall vividly the strange scenes I have witnessed over tickets in the dead of night; the unconscious traveller aroused from uneasy slumbers by a shout of "billets" in his ear; he jumps up alarmed at the brigand-like appearance of the guard, protected against the cold by a pointed capôte drawn over his head. The usual fruitless hunt commences, in which all the passengers join, languidly or vociferously, according to individual bias; every pocket is ransacked, every bag is inspected, the newspapers shaken out, the boots turned upside down, whilst everyone makes more or less imbecile suggestions. The guard at last, tired out, retires with an imperceptible smile hovering on his face, though he threatens untold penalties in an austere voice. The culprit feverishly continues to hunt, and when every package has been disturbed, and the whole carriage reduced to chaos, the missing ticket is discovered in the owner's waistcoat pocket, though he has already looked there three times.

*For Travel Advertisements see page xvii.*

### Royal British Nurses' Association.

THE first sessional lecture of the season was given last Friday at the rooms of the Medical Society of London by Miss Rosalind Pritchard. There was a good attendance of nurses, and amongst those present were: Mrs. Coster (honorary secretary), Miss Entwistle, Miss Thorold, and Sir Henry Burdett. Mr. Pickering Pick presided. The subject of the address was "Our Hospitals," and Miss Pritchard devoted herself chiefly to explaining the numerous and beautiful limelight views which illustrated it. At the close of the lecture an interesting discussion followed, in which Sir Henry Burdett (who kindly lent the photographic slides), Mr. Pickering Pick, Miss Entwistle, and Miss Thorold took part, on the advisability or otherwise of children in hospitals being housed in wards set apart for them.

### Christmas Books.

Christmas, as usual, has been a busy time for publishers, and this year has again seen the birth of many new books which will doubtless be appreciated in the leisure hours of the holidays. Among the many new ones that have just come out we suggest the following as likely to be interesting to our readers:—

A unique interest centres round Mr. Aubrey's little volume of "Strange Stories of the Hospitals"* since it is presented by the author as a free gift "to the Committee of the Hospital Saturday Fund, who will receive all profits arising from the sale of the book and apply them to the benefit of the hospitals and medical charities of London." There are five short stories comprised in the volume, among which "The Ambulance Corps Man" and "A Woman's Love" are the most interesting. The former is a humorous account of a retired railway porter, who, during his time in the company's service, had been an enthusiastic member of the Ambulance Corps. "Old Sammy had been one of the first to join an ambulance class, and had worked hard and enthusiastically throughout. He had his medals and certificates and—jointly with others in his 'corps'—had won prizes which had been presented amid the plaudits of select and appreciative witnesses." The "belief was that poor Old Sammy had worked at 'First Aid' till it had turned his brain; that an overmastering longing had taken possession of him—a yearning to put his knowledge into practice before his death. And it was hinted that, having never met with an opportunity of doing this, he now haunted the neighbourhood of the railway in a sort of half-crazy expectation that one day his services might be put in requisition." At last his opportunity occurs, and he gets a case on which to exercise his skill, and "He examined the prostrate and unresisting Mr. Curtis. He turned him this way and that on his face and on his back, looking very grave and wise the while; the driver and stoker watching all his movements, anxious and mystified, yet with a touching confidence in Old Sammy's well-known aptitude for 'First Aid' work. By degrees he bandaged Mr. Curtis up so that very little could be seen of him." . . . "A little later the senior surgeon at the hospital was examining, with much surprise and in some perplexity, a rolled-up bundle that had been brought into the accident ward, said to be a man who had been run over on the railway, but that quite as much resembled a mummy from an Egyptian tomb." Unfortunately for "Old Sammy" the patient turns out to have only been slightly stunned, and is more than indignant at his treatment. "'Let me get at that meddling old fool,' he said, struggling himself free of the bandages. But Old Sammy was already out of sight, and had left no trail behind!"

The story of an artist's world is "A Tragedy in Marble,"† whose hero, a sculptor, worked at art for his daily bread in his youthful days, but who later inherited a fortune, which brought with it leisure to pursue art in a *dilettante* fashion. In his days of poverty, in the heart of a Bohemian circle, Thornhill is shown as a happier man than in the affluent times which succeeded, and we follow his fortunes through these pages with interest, if with regret.

Sarah Tytler is again to the fore with a fresh novel, which will be good news to her many admirers. The scene of the present story, "Mrs. Carmichael's Goddesses,"‡ is laid in Dundee at the commencement of the century, before the numerous manufactories had arisen to spoil the picturesque appearance of the old Tayside town. Mrs. Carmichael, the principal figure in the story, is a lady whose husband has lost his money. She has an excellent business head and much common sense, and she at once starts in business as a cabinet maker. In this she is extremely successful, and when the story opens we find her a well-to-do widow with a ne'er-do-well and cast-off son and two daughters, the "goddesses" of the title. Although she enters heart and soul into her work, she will not permit her daughters to have anything to do with the shop, but keeps them entirely apart from all her commercial surroundings, her idea being that they shall marry into the class to which they belong both on their father's side and her own. Fate, however, wills it otherwise, and one of them marries David Wedderburn, the natural son of a neighbouring laird, while the other is won by a "stickit minister" and schoolmaster, the son of a small farmer. But in spite of her past ambition she is well satisfied with the way things turn out, and her final remark when the book closes is, "When all is done, I can say what many are not able to say, the Lord be thanked." The excitement of the story is furnished by the trial of her son for murder. He is proved not guilty through the timely appearance of the dominie with the real murderer's confession. In the end he marries a girl much beneath him socially, and starts for America, where he thinks he will be less likely to get into mischief, but he does not live to reach his destination. The ship goes down, and Charlie Carmichael loses his life in heroic and successful attempts to save those on board. The story is well and naturally told, and the picture of life in a small Scotch town a century ago shows equal skill and research.

A new book which we would specially urge our readers to buy and read for themselves is called "Prophets of the Century,"* and it is a book which should find a welcome place in the library of any thinking reader. The "Prophets" dealt with in these pages, which have been edited by Mr. Arthur Ricketts, comprise Wordsworth, Shelley, Thomas Carlyle, Ralph Waldo Emerson, Alfred Tennyson, Robert Browning, George Eliot, John Ruskin, Walt Whitman, William Morris, Leo Tolstoy, and Henrik Ibsen, a choice of names to which none could find exception, though we could have wished a few more had been added, since the field of Mr. Ricketts' "Prophets" is a cosmopolitan one, and not confined to England, which country, however, provides eight out of the dozen to which the editor directs our attention. In so far as this volume goes—and we are only suggesting that it might have gone a little further—it is one of extreme interest, and possesses a highly educational value by placing before the reader in a simple expository manner the teaching of those master spirits of the age, whose ideals have helped so largely to influence the minds of men in this country. Each of the essays comes from a different pen, and they are written by those who not only know their subject but know how to write about it. The name of only one woman—and that one George Eliot—figures in these pages as a prophet, and this delightful chapter on the work and teaching of one of the great literary "forces" of the nineteenth century is contributed by the editor.

A very fascinating edition of "The Vicar of Wakefield"† is just issued by Henry Frowde. This is the latest of the Oxford Miniature Thumb Editions; it is printed in perfectly legible type, 2⅛ by 1⅝ inches, on Oxford India paper, complete with collotype portrait, in 584 pages. What would the writer himself have said to this amazing publication of his immortal allegory? and, even amid the wonders of latter-day printing, it stands out as a perfect curiosity.

* "Strange Stories of the Hospitals." By Frank Aubrey. (London: Arthur Pearson. 1898. In paper, 1s.; in limp cloth, 2s. 6d.)

† "A Tragedy in Marble." By Adam Lilburn. (London: Chatto and Windus. 1898.)

‡ "Mrs. Carmichael's Goddesses." By Sarah Tytler. (London: Chatto and Windus. 1898.)

* "Prophets of the Century." (London: Ward, Lock, and Co. 1898. Edited by Arthur Ricketts. 6s.)

† The Oxford Thumb Edition of "The Vicar of Wakefield." (London Henry Frowde, Amen Corner, E.C. 1s.)

"My Horse, my Love,"* which originally appeared in America, is now for the first time offered, with considerable additions, to English readers. It gives, in the form of conversations with a Polish Count, the latter's views on horses in general and Arabs in particular. A good many of the views and opinions set forth will hardly be accepted by any one having any knowledge of horses, but the writer is evidently an enthusiastic lover of the equine species, and the book is written with the laudable object of obtaining better treatment for her favourites.

Tales of London life, instead of being extravagant descriptions of Belgravia and Mayfair, nowadays centre more often round the East-end—the poor East-end, with its many sorrows, and its few and fleeting joys—and it would appear there must be a certain satisfaction derived from the perusal of tales from the "far" East, if we can judge by the supply of such literature, which is ever on the increase. Of such a nature is "Slum Silhouettes,"† a collection of stories of London life by J. Dodsworth Brayshaw, who has woven into this series incidents many of which the author informs us were actual occurrences, and which illustrate his conclusion that "man is like a nut, not to be judged by the outer shell, but rather by that which lies within." They are clever little stories, and well worth reading. "In the Image of God"‡ also has "lower" London for its *mise-en-scène*. That Mr. Adcock writes from a personal knowledge of his subject is very evident, and that he is not unfamiliar with the tragedies that underlie the strata of society which is comprised in lower London is quickly seen. The hero and the heroine of the story are the hapless offspring of a drunken father, to whose neglect is due the death of their elder sister, the guardian angel of this home in the slums, from whose doors the wretched mother has absconded with some villain, whose name does not appear in Mr. Adcock's story. The boy 'Enry and the girl Ann, deserted by their father, are adopted —if one may stretch a point and call their future existence an adoption—by a relation, a certain Mrs. Loroff, an asthmatical, impoverished seamstress. The book is realistic, and no details of the children's career, whilst under Mrs. Loroff's "care," are spared one. But harrowing as is the description of their relation's "residence" (?), where carpet and curtains were ragged and full of dust, and where an air of discomfort and disorder brooded over everything, "yet to 'Enry and his sister the place was a comparative paradise. Mr. Guffin," the children's father, having comfortably transferred his parental responsibilities to Mrs. Loroff, vanished forthwith, and appears only at intervals throughout the pages—too often as a disturber of the children's peace, to use this term in its relative sense. There are various other characters in the book, each and all ably drawn, who live and speak as persons in such circumstances might be expected to do; it is an admirably written story, and 'Enry's troubles and his sister's, as also their stray and circumscribed joys, are very real to the reader. The children find a good friend in a working cobbler who resides on the ground floor of Mrs. Loroff's residence. His compassionate care for their welfare has its touching side. Later, at the close of the story, the unknown drama which the neighbours dimly hinted surrounded his life, is revealed, and discloses the reason of his care for the children. Towards the end of the story there is a chapter devoted to 'Enry's wooing, in which the young couple together assess their united earnings on which they intend starting housekeeping at 15s. per week. "It ain't much," Sue suggested. "It'll be eighteen when I'm on the job. We can furnish on the 'ire system. . . . We might put the banns up an' be tied up at the Register—say, next month; then you could stick on at the laundry a bit." It was not a glittering prospect, but it was good enough for Sue. "I'm game to chance it," she laughed, "if you are"; and 'Enry said he was. The wedding, however—for a description of which we would refer the reader to Mr. Adcock's book—is far from being on the simple lines as above dictated by 'Enry's prudence, but a roomy cab, flowers, rice, jeers, spectators, glasses of foaming beer at a passing public-house, &c, &c., raise the *tone* of the wedding scene. Anne, too, after a long apprenticeship in the hard school of want, finds a worthy mate, and the story has its happy bits; it would be pleasanter reading, though perhaps less dramatically true to life, had its writer not seen fit to wind up with a tragedy in which 'Enry and his wife, Sue, are the victims.

* By Sara Buckman Linard. (London: T. Fisher Unwin. 1898. Price 3s. 6d.)

† "Slum Silhouettes." By J. Dodsworth Brayshaw. (London: Chatto and Windus. 1898.)

‡ "In the Image of God." By St. John Adcock. (London: Skeffington and Son. 1898. 3s. 6d.)

### SOME CHRISTMAS MAGAZINES.

THE ENGLISH ILLUSTRATED MAGAZINE contains no less than 31 articles in the beautiful Christmas number issued for 1s.; and when such names as Maarten Maartens, E. Nesbit, and Edgar Jepson, Zack, Katharine Tynan, and Richard Pryce figure as writers, the literary merit of the publication speaks for itself. We find "The Surprise of Mr. Milberry," written by Jerome K. Jerome, is illustrated by Dudley Hardy, and this story in the opening pages of the magazine. Then there is an article on Eugenie, Daughter of Spain, Empress of France, exile in England, an article, which of necessity has a vein of sadness through it, and which is called "The Story of a Shadow," it is profusely illustrated with pictures of the Empress in the various stage of her chequered career; another article on "Women Duellists," by Colonel Willock; and on "Christmas Customs," &c. Some of the illustrations in this special issue of the *Englis Illustrated Magazine* are beautifully reproduced in colours, as is also the outside cover, which is a taking one.

CASSELL'S MAGAZINE.—The grand Christmas number of the above magazine may fairly be said to surpass any previous issue. It contains the opening chapters of Max Pemberton's "Garden of Swords," complete stories by Ouida, Anthony Hope, Robert Barr, William Le Queux, Guy Boothby, &c. A magnificent presentation plate is also included, and the magazine is full of charming pictures reproduced in colour. Besides the attractive stories by the above authors, there are a great number of articles likely to prove of general interest to the reading public, among which must be mentioned "How Her Majesty and the Royal Family Spend Christmas," by Miss Spencer Warren; the paper is abundantly illustrated. Then there is "Christmas in the Army," by the Rev. E. J. Hardy, Chaplain to the Forces, which relates many particulars of the manner in which Tommy Atkins keeps the festive season. Mrs. Tooley writes at some length on Sarah Bernhardt, and gives numerous illustrations of the great actress in her private life, and gives some pictures of her work as a sculptor.

THE BOY'S OWN PAPER.—The special Christmas number of the *Boy's Own Paper* has very charming contents, under an attractive cover. There are endless drawings and sketches and articles on subjects which are of interest to boys, and, taken altogether, this is as fine a sixpennyworth of reading as any boy could possess himself of in the space of one magazine.

### "The Hospital" Convalescent Fund.

WE have received the following letter and donation from the Cargona Company (Limited), of Cross Street, Peterborough: "We beg to enclose a cheque for 10s. 6d. as a small contribution towards your Convalescent Fund. Our lozenges are at all times at the free disposal of convalescents."

### Help the Nurses to Help the Sick.

**Royal National Pension Fund for Nurses** 28, Finsbury Pavement, E.C.—This Fund has during the past year maintained the uninterrupted success which has attended it ever since its establishment. The number of new members who have joined the Pension Fund in 1898 has been considerably above the yearly average. Nearly £1,500 has been distributed in sick pay, a fact which speaks eloquently as to the blessing which this branch of the Fund's work must be to nurses, more especially, of course, to those working on their own account. More than £2,500 was paid away in pensions and bonuses, against £1,773 in 1897, £1,279 in 1896, and £817 in 1895, showing how greatly the Fund is increasing its sphere of usefulness. The premium income, *i.e.*, the payment by or for nurses, exceeded the enormous total of £65,000.

**The Junius S. Morgan Benevolent Fund** is an auxiliary to the above fund for nurses, was founded through generous contributions from nurses themselves, and raised to handsome proportions by the munificence of the Morgan family and many other friends to nurses. The work is done by volunteers, under the supervision of an influential committee, which devotes time and care to the investigation of claims and the relief of urgent cases. Hon. Secretary, Miss Rosalind Pritchard.

**East London Nursing Society.**—The object of this society is to nurse the sick poor in East London in their own homes by means of trained resident nurses, each nurse living in the parish in which she works. The extent of the society's useful work is shown by the fact that in 1897 the staff of 33 nurses attended to 5,809 persons, to whom 127,032 visits were made. Annual subscriptions and donations to the general fund are asked for. Secretary, Mr. Arthur W. Lacey, 49, Philpot Street, Commercial Road, E.

**Metropolitan Nursing Association**, Bloomsbury Square, W.C.—Founded in 1875 as a Training School and Home for District Nurses who have previously gone through a full course of hospital training, and who nurse the sick poor in their own homes within a radius of a mile and a half from Bloomsbury Square. This Association is now the central training home for the Queen's Nurses. Superintendent, Miss E. B. G. Gray.

**Queen Victoria's Jubilee Institute for Nurses.** Offices: St. Katharine's Precincts, Gloucester Gate, Regent's Park, N.W.—The institute trains nurses in district nursing, and supplies nurses for the sick poor in their own homes. Applications for information should be addressed to Miss Peter, the Inspector. Nursing associations in Scotland, Ireland, and Wales are affiliated with the institute.

**"The Hospital" Convalescent Fund.**—Since the establishment of this fund many tired and delicate workers have enjoyed a much-needed change of air such as they could not possibly have secured for themselves without help. Experience has proved that it is better to let the nurses have a choice of locality rather than to send them to one settled place, and the object of the fund, namely, to provide rest for weary workers, amidst suitable surroundings, without any of that anxiety about ways and means which retards convalescence is fully carried out. Contributions which would increase the field of usefulness are invited by the Hon. Secretaries, care of the Editor of THE HOSPITAL.

**Up-Country Nursing Association for Europeans in India.**—The chief object of this association is the provision of skilled nursing for Europeans, especially civilians, in the up-country districts of India. The association in London selects the nurses, and pay all their expenses until they arrive at their destination in India. Once established there, the cost of maintaining the nurses falls upon the Local Committee. Hon. Secretary, Major-General J. Bonus, R.E., The Cedars, Strawberry Hill.

**The Colonial Nursing Association**, the Imperial Institute, S.W.—This valuable association was founded two years ago to supply trained nurses in the Crown Colonies. It is one that appeals to the sympathies of all, for which family is there that has not some members in distant lands, building up the Empire, and fighting with the sickness that comes with rough faring and undrained country? The Hon. Secretary, Miss Piggott, will be glad to receive contributions, especially as an effort is being made just now to extend its benefits to the poorer colonies.

### Everybody's Opinion.

[Correspondence on all subjects is invited, but we cannot in any way be responsible for the opinions expressed by our correspondents. No communication can be entertained if the name and address of the correspondent is not given, as a guarantee of good faith but not necessarily for publication, or unless one side of the paper only is written on.]

### BATHS IN TYPHOID FEVER.

"A TRAINED NURSE," writing from Rome, says: The excellent results obtained by the cold bath treatment for typhoid fevers have again been seen during these last months in Rome. In one of its large hospitals, out of 152 cases treated in this way, only four were lost. Amongst those who recovered were patients who had suffered from every possible complication, often prolonging the need for antipyretic treatment to the number of seventy or eighty baths. Pneumonia, nephritis, and meningitis were all combatted successfully, whilst one patient even survived perforation! This last complication took place during the night; the patient was immediately wheeled to the theatre and operated by the surgeon in charge, recovering afterwards perfectly. The baths are given, almost invariably, when the patient's temperature reaches 39·5 deg. C (103 deg. F.) The tubs are wheeled to the bedside, the water prepared at 28 deg. C., and gradually reduced to 25 deg. C., the patient remaining in the bath from fifteen to twenty minutes.

### NURSING INSTITUTES AND THEIR NURSES.

WE publish an extract from an impudent letter received by us, which, so far as it shows the efforts that are made by the managers of some nursing institutes to suppress criticism, should be of much interest to nurses. As regards the threat of "immediate action" contained in this letter, we have only this to say, that if nurses find that they are deceived or imposed upon by managers of nursing institutes, no such threats as those made by the writer of the following letter will in any way prevent us from giving full publicity to our opinion on the subject:—

"Nursing Institute and Medical Home, West View,
"22, Claremont Road, Cricklewood, N.W.

"SIR,—I wish to inform you that Nurse ——, but who styles herself 'Sister ——,' threatens to write an article to THE HOSPITAL because I requested her to leave the institution unless she was prepared to take a case. If anything is published mentioning, directly or indirectly, my name, address, or anything appertaining to me or the institution, I have instructed my solicitor to take immediate action against the proprietors of THE HOSPITAL paper and against the nurse. . . .—I am, faithfully yours,
"A. J. ELLIOTT, Matron."

### "A WORD OF WARNING."

THE SECRETARY OF THE INTERNATIONAL COUNCIL OF WOMEN writes: I am directed to reply to a paragraph which appeared in THE HOSPITAL of November 5th, copies of which you were good enough to forward to members of the Committee of Arrangements for the International Congress of Women to be held in London next June. The paragraph is headed, "A Word of Warning," and criticises the preliminary arrangements of the nursing section of the Congress. I beg to point out to your readers that the names of speakers mentioned at the meeting at Norwich of the National Council of Great Britain and Ireland were those invited by the International Executive, which met in London last July; to these were added the names of speakers proposed by the National Council of this country, each affiliated National Council having the right to send in such a list of names for the approval of the Committee of Arrangements, who have been entrusted with the carrying out of the details of the forthcoming Congress. The Committee of Arrangements is anxious that every branch of women's work shall be adequately represented, and to attain this desirable object the work of preparation has been divided among various sectional committees. For instance, medicine, midwifery, and nursing will be dealt with in the professional section, of which Mrs. Bedford Fenwick has been appointed the convener. For the nursing profession the following well-known ladies have been invited to take seats on the Professional Section Committee: Miss Isla Stewart, matron of St. Bartholomew's Hospital, who has accepted; Miss Gordon, matron of St. Thomas's Hospital, who has declined on the ground of being too fully occupied; Miss Monk, matron of King's College Hospital; Miss Pauline Peter, Inspector of Queen's Nurses; Miss Gibson, matron of the New Infirmary, Birmingham; Mrs. Strong, matron of the Royal Infirmary, Glasgow; and Miss M. Hurley, matron of Sir Patrick Dun's Hospital, Dublin. Miss Louisa Stevenson, a member of the Board of Management of the Edinburgh Royal Infirmary, has consented to act on this committee. The Committee of Arrangements hope that the leaders of the nursing profession will give them cordial support in making the nursing conference a success, and they will, no doubt, later on communicate with the matrons of the chief hospitals, infirmaries, and nursing institutions of this country inviting their kind co-operation. I may add that already intimation has been received of the desire of representative nurses in many parts of the world to attend this congress.

*** We repeat our advice that British nurses should take care that they are properly represented at the coming Congress. A serious responsibility attaches to leaders in the nursing world if they fail to take their proper place on this occasion.—Ed. *T. H.*

### Minor Appointments.

Brook Hospital, Shooter's Hill, S E —Miss Annie Thomas has been promoted from charge nurse to Night Sister of the above. She received her training at the General Hospital, Birmingham.

Lanchester Workhouse Infirmary.—On December 1st Miss Ellen Watson was appointed Superintendent Nurse of this infirmary. She was trained at the Union Infirmary, Salford, at which place she was afterwards charge nurse.

Eastville Workhouse Hospital, Bristol.—Miss Mary Griffiths has been appointed Night Sister of this institution. She was trained at Hants County Hospital, and afterwards was assistant nurse at the Bristol Royal Infirmary. Miss Griffiths is now charge nurse of the Infirmary, Burton-on-Trent.

The Park Hospital, Hither Green, S.E.—The following Charge Nurses have recently been appointed here: Miss Mary Gwens; nursing since 1883; trained at the Adelaide Hospital, Dublin; since held posts as charge nurse at Polytechnic Hospital, New York, and St John's Hospital, Brooklyn, N.Y. Miss May Matilda Dods; nursing since 1894; trained at Lewisham Infirmary, S.E.; since charge nurse at Chelsea Hospital for Women. Miss Jessie Hancock; nursing since 1892; trained at Colchester Infirmary; assistant nurse, South-Western Hospital, 1892-1895. Miss Sara Hackney; nursing since 1885; trained at Royal Hospital, Belfast; since charge nurse, Royal Infirmary, Edinburgh, and private nursing, Hanover Institute. Miss Ellen Ringrose; nursing since 1891; trained at South Devon Hospital, and Infirmary, St. George-in-the-East; charge nurse at Infirmary, St. George-in-the-East; superintendent nurse at Chertsey Infirmary. Miss N. F. Wheeler; nursing since 1896; trained at General Hospital, Birmingham, and Royal Hospital, Richmond. Miss F. M. de Blaguiere; nursing since 1894; trained at St. Vincent's Hospital, Dublin. Miss Kathleen Murphy; nursing since 1893; trained at Mater Misericordia Hospital, Dublin; since private nursing.

### Appointments.

Ashton-under-Lyne District Nursing Association.—On October 17th, 1898, Miss Aldis was elected Superintendent of this association. She was trained in the General Hospital, Weston-super-Mare, and she has successfully held the following posts: Staff nurse at the City of London Chest Hospital; district and private nurse, Bishop's Stortford Institute; sister, Essex and Colchester Hospital; matron, Fever Hospital, Stratford-on-Avon; lady superintendent and lecturer, the County Home for Nurses, Blandford, Dorset; and temporary lady superintendent, Bolton Private Nurses' Institute.

Birmingham and Midland Counties Training Institute for Nurses.—Miss M. D. W. Ewing, who was trained at the Children's Hospital, Glasgow, and at the Royal Infirmary, Edinburgh, was appointed on the 6th inst. Lady Superintendent of this institution. Her previous appointments have been as follows: Ward sister and night superintendent at the Aberdeen Royal Infirmary; sister in charge at the Aberdeen Children's Hospital; and for six years lady superintendent of the Broomhill Home for Incurables, Glasgow.

Royal Cornwall Infirmary, Truro.—Miss A. Blanche Trew has been appointed Matron of this infirmary, and enters upon her duties on the 22nd inst. Miss Trew was trained at University College Hospital; was staff nurse at the Children's Hospital, Paddington; ward sister at the General Infirmary, Derby; staff nurse at the National Hospital for the Paralysed and Epileptic; home sister and assistant lady superintendent of the Nurses' Co-operation, 8, New Cavendish Street, W., of which association she has been a member for six years.

Hereford General Infirmary.—On the 8th inst. Miss Katherine Elphick, who was trained at King's College Hospital, was appointed Matron of this institution. She has been sister at the General Hospital for Children, Pendlebury, Manchester; sister at the Hospital for Sick Children, Great Ormond Street; and lady superintendent of the North London Hospital for Consumption and Diseases of the Chest.

Cambridge Sanatorium for Infectious Diseases.—Miss M. A. Isabel Thomas was selected on the 10th inst. Matron of the above institution. She was trained at Grantham Hospital, and has held the posts of staff nurse at Monsall Fever Hospital, sister at Hull Fever Hospital, matron of the Hull Small-pox Hospital, and senior sister at the Aberdeen City Hospital.

Derby Borough Asylum.—Miss Kate Barwick has been appointed Matron of the above institution. Miss Barwick was trained at the Nottingham Infirmary, and subsequently at James Murray's Royal Asylum, Perth, and latterly acted for two years and a half as staff nurse at the Perth District Asylum, Murthly.

The Hospital, Bermuda.—The new Matron, who was appointed here on November 12th, 1898, is Miss Elizabeth M. Crawford. She was trained at Westminster Hospital, where she held the post of staff nurse in several wards, and where for three years she was sister-in charge of the outpatient department.

Whitworth Hospital, Darley Dale, near Matlock.—The name of the new Matron here is Hoyle, not Boyle, as stated last week.

### For Reading to the Sick.

### CHRISTMASTIDE.

"GLORY to God in the highest, and on earth peace, good will toward men."

**Verses:**

Let not the hearts, whose sorrows cannot call
This Christmas merry, slight the festival;
Let us be merry that may merry be,
But let us not forget that many mourn;
The smiling Baby came to give us glee,
But for the weepers was the Saviour born.
—*H. Coleridge.*

Blest day which aye reminds us year by year
What 'tis to be a man; to curb and spurn
The tyrant in us; that ignobler self
Which owns no good save ease, no ill save pain,
No purpose save its share in that wild war
In which through countless ages living things
Compete in internecine greed!
While ever out of the eternal heavens
Looks patient down the great magnanimous God,
Who, Maker of all worlds, did sacrifice—
All to Himself? Nay, but Himself to One;
Who taught mankind on that first Christmas Day
What 'twas to be a man; to give, not take,
To serve, not rule; to nourish, not devour;
To help, not crush; if need, to die, not live!
—*Kingsley.*

Thou cam'st from heaven to earth that we
Might go from earth to heaven with Thee,
And though Thou found'st no welcome here
Thou did'st provide us mansions there.
—*H. Vaughan.*

Immanuel! God with us in His meekness;
Immanuel! God with us in His might,
To bind our wounds, to gift with strength our weakness,
To bring us, angels, to the home of light!
Shiloh is come; His feet our earth have trod;
Now thanks and glory to the Child our God.
—*Morgan.*

What is man, that Thou art mindful of him? and the son of man that Thou visitest him?—*Ps.* vii. 4.

**Reading.**

"Suddenly there was with the angel a multitude of the heavenly host praising God . . ."—*St. Luke* ii. 13.

Three times are we told in Scripture that the angels sang. At the birth of the world, when the foundations of the earth were laid, the morning stars sang together and all the Sons of God shouted for joy. When Jesus was born into the world a multitude of the heavenly host praised God, and said, "Glory to God in the highest, and on earth peace, good will towards men." The angels had a clear perception of the purpose of Christ's coming. One of the chief of them said to Joseph, "Thou shalt call His name Jesus: for He shall save His people from their sins." And they all sang when he came, because they knew that God was now dealing in a special and most effective way with that dark thing which cast its shadow on heaven as well as on earth. And when anyone is born again there is joy among the angels in heaven over the sinner that repenteth. The subject of the song in each case is the same; the leading *motif* of them all is man. Man, to begin with, was God's chief end in creation, and the angels sang not so much because a new world had been made, but rather because a new being akin to themselves was put into it to whom they might minister and with whom they might co-operate in the doing of God's most holy will; and this season comes to remind us of our inherent dignity in God's sight, of the noble ideal He has formed for us, of the value He sets on those whom He sent His Son to seek and to save. —*Rev. Hugh Miller.*

### Notes and Queries.

**The contents of the Editor's Letter-box have now reached such unwieldy proportions that it has become necessary to establish a hard and fast rule regarding Answers to Correspondents. In future, all questions requiring replies will continue to be answered in this column without any fee. If an answer is required by letter, a fee of half-a-crown must be enclosed with the note containing the enquiry. We are always pleased to help our numerous correspondents to the fullest extent, and we can trust them to sympathise in the overwhelming amount of writing which makes the new rules a necessity.**

**Every communication must be accompanied by the writer's name and address, otherwise it will receive no attention.**

*Free Convalescent Home.*

(122) Can you tell me if there is a convalescent home, or home where a patient with chronic bronchitis could be received for a month or two without payment? Yorkshire or Lancashire preferred.—*Nurse Kirk.*

See list in "Burdett's Hospitals and Charities," 5s., post free, from Scientific Press, London. Possibly the Southport Convalescent Hospital, by a subscriber's letter, might suit.

*General Training.*

(123) Please inform me if there are institutions where I could get a few months' training in general nursing. I am a certificated masseuse. Would give my services or would pay. Age 33.—*M. G.*

It is difficult to obtain general training on reciprocal terms. Many of the general hospitals would receive you as a paying probationer. See Burdett's "Official Nursing Directory" (Scientific Press, 5s.), for lists.

*The Youngest Probationer.*

(124) What is the earliest age that a probationer can be taken in any children's hospital?—*N. P. A.*

Ulster Hospital for Children, Belfast, receives probationers at the age of 18. The usual age at other hospitals is 20 or 21. See Burdett's "Official Nursing Directory" (The Scientific Press, price 5s.)

*Hertford Hospital, Paris.*

(125) Will you kindly give me all the information you can respecting the English Hospital in Paris? Is it easy to get an appointment there? I have had one year's training, and wish for more.

Write direct to Matron, Hertford British Hospital, Rue de Villiers, Levallois-Perret, Paris.

*Acute Mania.*

(126) Can any of your readers tell me of a free home where a child of four years of age suffering from acute mania can be received? I am told he is too young for ordinary lunatic asylums.—*Matron.*

Write to the medical superintendent of your county asylum.

*Appointment Abroad.*

(127) Would you kindly inform me how I could enter a Government hospital abroad?—*Nurse H.*

Apply the Colonial Nursing Association, The Imperial Institute, London, and see our advertisement columns.

*Convalescent Home in Edinburgh.*

(128) I should be more than grateful if you could inform me whether or no there is a convalescent home, or a home of any kind where paying convalescents would be received, in the neighbourhood of Edinburgh. I have sent a stamp, as I have a particular objection to having a post-card in reply.—*M. C.* (*Wigan*).

"M. C." has failed to read our rules to correspondents. Any letter requiring an answer by post must be accompanied by a fee of 2s. 6d. Miss Cole, 188, Ebury Street, London, S.W., will forward a list of holiday and convalescent homes for 1½d.

*Male Nurses.*

(129) 1. Will you kindly tell me where men nurses are trained. I am 26 years of age, and should like to know (2) if I am the right age, how long I should have to give to probation, and (3) if there would be a premium?—*W. P.*

1. Male nurses are trained in England only at the National Hospital for the Paralysed and Epileptic, Queen's Square, Bloomsbury. Write to the matron for information. 2. Your age is suitable. Some of the larger lunatic asylums afford good training for male nurses who are willing to take up mental work first and the general nursing afterwards. 3. There is no premium.

*Hard Up.*

(130) Can you tell me if there is any way in which I could earn a little money? I am a certificated nurse, but for the last two years have had rheumatic neuritis in my left arm, and can only use my thumb and first finger a very little, as the rest of my fingers, also shoulder and elbow are so stiff.—*B. C.*

Can our readers help?

*Holiday Wage.*

(131) Nurse "E. A. B." has been 14 months with a patient, fee £2 2s. per week, paid monthly; she is to have a week's holiday at Christmas. Would it be considered right to charge for that week when sending in her next account afterwards? No arrangement was made about holidays.

As no arrangement was made the payment could not be enforced. Would not this be a good opportunity to talk over the matter of holidays in a friendly way with your employer?

*St. Paul's Cray.*

(132) Will you kindly tell me what you know of St. Paul's Cray Cottage Hospital?—*C. M.*

St. Paul's Cray Cottage Hospital has 15 beds, is nursed by a matron, one nurse, and two probationers. The certificate is for two years, and the probationers receive no salary. Premium £15. Private replies to letters are only given on receipt of a 2s. 6d. fee.

## Figures and Tables

**Figure f1:**